# UV-Vis Spectroscopy: A New Approach for Assessing the Color Index of Transformer Insulating Oil

**DOI:** 10.3390/s18072175

**Published:** 2018-07-06

**Authors:** Yang Sing Leong, Pin Jern Ker, M. Z. Jamaludin, Saifuddin M. Nomanbhay, Aiman Ismail, Fairuz Abdullah, Hui Mun Looe, Chin Kim Lo

**Affiliations:** 1 Institute of Power Engineering, College of Engineering, Universiti Tenaga Nasional, Kajang 43000, Selangor, Malaysia; ysleong@uniten.edu.my (Y.S.L.); mdzaini@uniten.edu.my (M.Z.J.); saifuddin@uniten.edu.my (S.M.N.); Aiman@uniten.edu.my (A.I.); fairuz@uniten.edu.my (F.A.); 2 Tenaga Nasional Berhad (TNB) Research Sdn. Bhd., Bandar Baru Bangi, Kajang 43000, Selangor, Malaysia; hui.mun@tnb.com.my (H.M.L.); chin.kim@tnb.com.my (C.K.L.)

**Keywords:** color, oil insulation, power transformers, ultraviolet-visible spectroscopy

## Abstract

Monitoring the condition of transformer oil is considered to be one of the preventive maintenance measures and it is very critical in ensuring the safety as well as optimal performance of the equipment. Various oil properties and contents in oil can be monitored such as acidity, furanic compounds and color. The current method is used to determine the color index (*CI*) of transformer oil produces an error of 0.5 in measurement, has high risk of human handling error, additional expense such as sampling and transportations, and limited samples can be measured per day due to safety and health reasons. Therefore, this work proposes the determination of *CI* of transformer oil using ultraviolet-to-visible (UV-Vis) spectroscopy. Results show a good correlation between the *CI* of transformer oil and the absorbance spectral responses of oils from 300 nm to 700 nm. Modeled equations were developed to relate the *CI* of the oil with the cutoff wavelength and absorbance, and with the area under the curve from 360 nm to 600 nm. These equations were verified with another set of oil samples. The equation that describes the relationship between cutoff wavelength, absorbance and *CI* of the oil shows higher accuracy with root mean square error (*RMSE*) of 0.1961.

## 1. Introduction

Power transformer is one of the main components in any transmission networks and they are responsible for transmitting electric power over long distances with minimal power loss. It is essential for power transformer to operate optimally in order to provide continuous and stable electricity supply in assuring homes and businesses run smoothly with minimum disruptions. Any failures in power transformers could lead to huge problems to both consumers and the power utilities. Therefore, it is important to carefully maintain, inspect, and monitor the power transformer over times.

The lifetime of power transformer is generally related to the degradation of insulating materials in power transformer. Although various sensors such as temperature sensor, pressure sensor, and humidity sensor are installed for online monitoring, the output of these sensors cannot provide clear indication on the conditions of the power transformer. One of the insulating materials is the transformer oil which acts as an electrical insulator and also a coolant for the power transformer. After years of services, the transformer oil is subjected to thermal and electrical stresses [1,2,3], hence causing oxidative stress on the oil. With the presence of oxygen and moisture [4], polar compounds and oil sludge are formed [5], and these oxidative by-products affect the quality of the transformer oil. The quality of the oil can be reflected on the properties of the oil and its content, such as acidity, dielectric breakdown voltage, dissolved gases, and furanic compounds [6]. Thus, many diagnostic methods were introduced and are capable of providing reliable assessment on the condition of power transformer in order to detect early faults and avoid potential failures [7].

Conventionally, the quality of the transformer oil is assessed based on its properties using IEC 60422 standard [6]. Various properties are tested with different techniques. For instance, dissolved gases are conventionally measured using gas chromatography (GC) [8], and interfacial tension of the oil is typically measured using the Ring method [9]. Generally, these techniques involve complex sample preparation, tedious measurement procedures, expensive instruments, and require trained experts to conduct the measurements. Thus, researchers are proposing alternative solution to assess the quality of the oil. Differential Scanning Calorimetry (DSC) method [10], dielectric dissipation factor measurements [11], infrared (IR) spectroscopy [11], capacitance measurement [12], and photoluminescence spectroscopy [13] have been proposed recently to evaluate the degradation of the transformer oil. Recently, as an attractive and promising analytical tool, the optical spectroscopy technique starts to emerge in assessing the degradation of transformer oil.

Optical spectroscopy studies the interaction of light by matter [14]. It is a non-destructive test, which provides rapid result analysis, and it can be used for qualitative and quantitative analysis [15]. The technique also gives high sensitivity measurements and does not require complex sample preparation and calibration. For instance, Deepa et al. [16] uses multidimensional fluorescence techniques like synchronous fluorescence spectroscopy (SFS) and excitation emission matrix fluorescence (EEMF) to determine the unique characteristic of degraded transformer oil in the spectrum. Kamenchuk et al. [17] have also applied nuclear magnetic resonance (NMR) in determining the chemical composition of the degraded oils. To provide complementary interpretations and full product description, Godinho et al. [18] combined data from three different optical sensing techniques, which are the near-infrared (NIR), EEMF, and NMR spectroscopy, to evaluate the quality of the transformer oil. Bakar et al. [9] have also proposed to measure the interfacial tension of transformer oil using ultraviolet-to-visible (UV-Vis) spectroscopy which reflects the insulation aging activity in the power transformer. Hussain et al. [19] have also made an evaluation of the state of the transformer oil using the combination of UV-Vis, Fourier transformed infrared (FTIR), and NMR spectroscopy techniques. Results show that each sensing technique is able to provide different analysis in determining the degradation level of the transformer oil.

To assess the degradation level of transformer oil using optical sensing techniques, researchers either determines the special characteristic of degraded transformer oil in the spectrum [16], or the change in concentration of certain content in transformer oil, such as inhibitor content [17] and furanic compounds [18], or the change in properties of transformer oil such as interfacial tension [20]. The color of the transformer oil can also be used to assess the degradation level of the transformer oil [7,21]. Conventionally, using the American Society for Testing and Materials (ASTM) D 1500 standard (Standard test method for ASTM Color of Petroleum Product (ASTM Color Scale) ) [22], the color of the transformer oil is determined using a color comparator, and represented by a color index (*CI*) from a scale. The scale contains 16 ASTM *CI*, with increment in steps of 0.5, starting from 0.5 for the lightest color to 8.0 for the darkest color. However, this method has its disadvantages. Firstly, the step size of 0.5 *CI* is fairly large. If an exact match is not found for the sample, the darker of the two ASTM colors is reported instead. In many cases, the sample does not fall exactly on the 16 values used in the ASTM D 1500. Secondly, this method relies on the manual visual inspection by an operator and the results may also be affected by the quality of standard light source. Different operators working on an identical oil sample could report a different *CI*. Likewise, in color measurement of palm oil, an operator is also required to manually operate Lovibond® Tintometer [23] to determine the right color index of the palm oil. Subsequently, operators that carry out this measurement also need to go for eye checking every 3–6 months due to safety and health issues. According to occupational safety and health guideline, long exposure time with high intensity of visible light on the eye could pose health issues such as headache and eye-related problems. Finally, this issue leads to limited number of samples per day for color index analysis as the operator is required to rest their eyes after testing each sample.

Hence, a more accurate, scientific and automated method in observing the color of the oil is needed. Therefore, this paper proposes to use UV-Vis spectroscopy as an optical sensing technique to determine the color index of transformer oil. The application of UV-Vis spectroscopy in sensing the color of oil dated as early as 1999 where Chantrapornchai et al [24] studied on the spectral reflectance and color of oil-in-water emulsions. Tan et al. [23] have proposed a prototype colorimeter that utilizes UV-Vis spectroscopy to determine the color of palm oil and made comparison between their propose method and the conventional method. UV-Vis spectroscopy also has been widely used in evaluating olive oil since the components within such as chlorophyll and lutein are responsible for the color of olive oil [25,26]. Besides palm oil and olive oil, researchers have also applied the UV-Vis spectroscopy in determining the color in crude petroleum oil [27], hydraulic oil [28], automotive lubricating oil [29], essential oil [30], pumpkin seed oil [31], and edible oils (sunflower oil, soya oil, corn oil, canola oil, and olive oil) [32]. Moreover, Hadjadj et al. [21] have proposed the determination of the correlation between proposed parameters, which are turbidity and dissolved decay product (DDP) value, and the traditional parameters such as interfacial tension, acidity and color index of transformer oil using UV-Vis spectroscopy. Results show a good correlation between the parameters, but the color index is instead based on the 18 Gardner color scale in accordance with ASTM D 1544 [21]. We have also demonstrated the possibility of determining the *CI* of transformer oil based on ASTM D 1500 using UV-Vis spectroscopy [33]. In this work, the utilization of UV-Vis spectroscopy to determine the *CI* of transformer oil is fully established through a detailed study on the optical spectral response of oil samples with different *CI*. Furthermore, mathematical models that describe the relationship between the optical response of the oil sample and its respective *CI* were formulated and verified.

## 2. Materials and Methods

### 2.1. Sampling and Sample Design

For the purpose of this study, transformer oil samples were sampled from different operating transformers. The transformer oil used in these power transformers are generally naphthenic-based transformer oil without additives (uninhibited), produced by Hyrax, Petronas or Shell. The transformer oil product typically contains 55% naphthenic carbons, 38% paraffinic carbons and 7% aromatic carbons, and it fulfills the performance requirement set by the (International Electrotechnical Commission) IEC 60296 standard. The procedure of collecting and transporting these samples adhere strictly to the IEC 60475 standard [34]. This is to ensure that there was no contamination and mislabeled of the oil samples that would affect the analysis. By taking this into account, two 1 litre amber glass bottles were prepared for transformer oil sampling. One bottle of the sample was sent to an accredited lab for conventional analysis, while the other bottle was used for the purpose of this study. This was done to guarantee that there was minimal time gap between the conventional lab analysis and the experiment of this study. In accordance to the ASTM D 1500 standard, the conventional analysis involves a color comparator to measure the color index of oil samples and report them with one of the 16 ASTM color index. Throughout this paper, the color index measurement carried out at the accredited lab in accordance to the ASTM D 1500 standard will be referred to as conventional analysis or conventional measurement.

### 2.2. Optical Measurement Setup

The samples sent for the purpose of this study were measured for their absorbance spectrum to the range of 200 nm to 800 nm. The measurements were carried out using Agilent Cary 5000 Ultraviolet-to-Visible-to-Near-Infrared (UV-Vis-NIR) spectrophotometer (Agilent Technologies, Petaling Jaya, Malaysia). The Cary 5000 is a double beam spectrophotometer that can operate in the range of 200 nm to 3300 nm. The general operation of a double spectrophotometer can be found in [35]. Approximately 3 mL of oil sample and clean uninhibited transformer oil are transferred using a 5-mL disposable plastic pipette into quartz cuvette which has an outer cell dimension of 12 mm × 12 mm × 44 mm, with an optical path-length of 10 mm. The clean oil serves as the reference for the measurement. For each oil sample, the optical spectrum measurements were repeated 3 times to ensure its consistency and repeatability. During the spectrum scan, the light beam passes through both oil sample and reference sequentially and optical transmittance was recorded. The absorbance values were calculated by applying the Beer-Lambert Law [36] as in Equation (1).

*Abs* = −log_10_(*S_λ_* − *B_λ_* / *R_λ_* − *B_λ_*) = *ε_λ_* • *c* • *l*, (1)

where, *Abs* is the absorbance, *S_λ_* is the transmittance of light passing through the sample in sampling slot, *R_λ_* is the transmittance of light passing through the sample in reference slot, *B_λ_* is the baseline, *ε_λ_* is the absorbance coefficient of the absorbing sample at a certain wavelength, *c* is the concentration of the absorbing sample, and *l* is the path-length traversed by the light.

### 2.3. Initial Results and Modification

**Figure 1:**
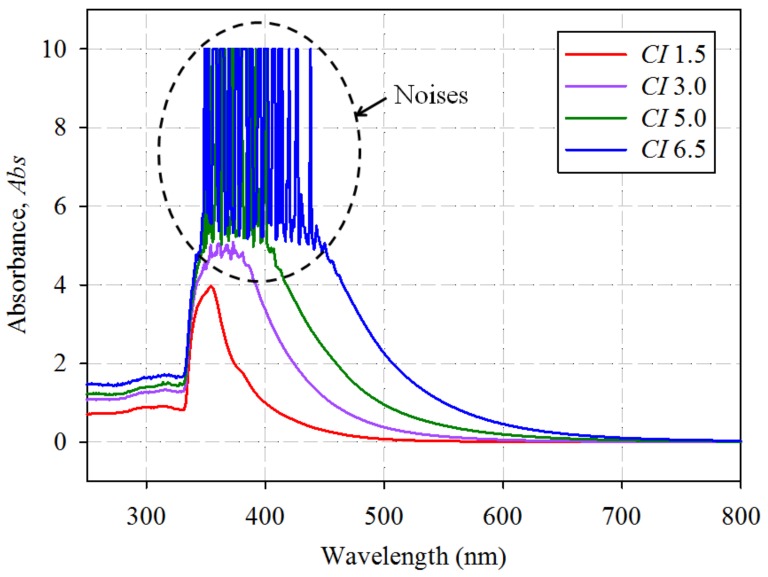
Initial optical absorbance spectrums of four oil samples with different color indices (*CI*) based on ASTM D 1500.

Figure 1 shows the absorbance spectral response together with the noise of oil samples with *CI* of 1.5, 3.0, 5.0 and 6.5.

**Figure 2:**
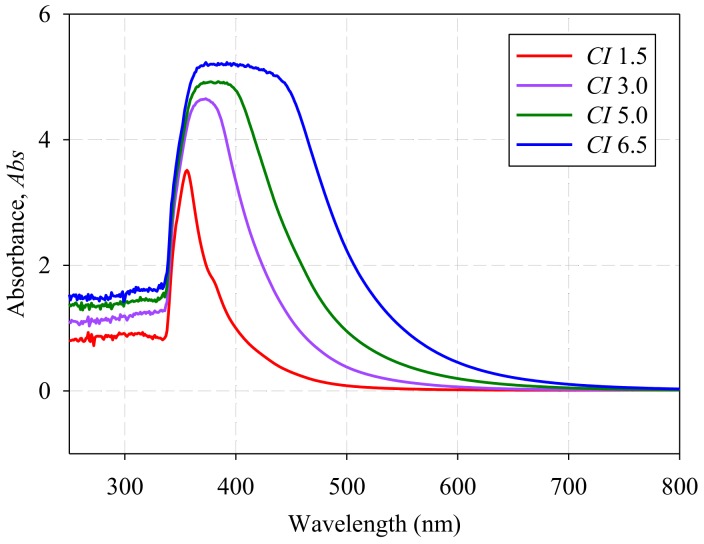
Optical absorbance spectrums of four oil samples with different *CI* based on ASTM D 1500 after applying ND filter.

Initial results show that noises can be observed at the peak absorbance of the oil samples with *CI* of 3.0 or higher, and the spectral response saturates at *Abs* > 5. Referring to Equation (1), if the ratio of *S_λ_* to *R_λ_* is extremely low, *Abs* will be extremely high, resulting in noises due to the fluctuation of *S_λ_*. In order to increase the ratio of *S_λ_* to *R_λ_*, *S_λ_* has to be increased or *R_λ_* has to be decreased. *S_λ_* cannot be increased further as the brightness of the light source in the spectrometer affects both sample and reference sides. Therefore, *R_λ_* needs to be decreased to remove the noises. To reduce *R_λ_*, a neutral density (ND) filter (model: FNDU-20C02-0.1), which provides significant optical attenuation (Transmittance = 1%–3%) in the UV-Vis region, was applied at the reference slot of the spectrometer. The noises can also be reduced by decreasing *l*, which means a shorter path-length cuvette is used. However this method reduces the interaction of light with the oil samples, thus reducing the sensitivity of the measurement. Due to the shortcomings of a shorter path-length, the ND filter was chosen to reduce the measurement noise. The outcome is shown in Figure 2, where the noises at the peaks (top) of absorbance spectral response of the same four oil samples in Figure 1, are eliminated and clear smooth optical absorbance spectrum can be observed.

## 3. Final Results and Data Analysis

### 3.1. Absorbance Spectra of Transformer Oil Samples

**Figure 3:**
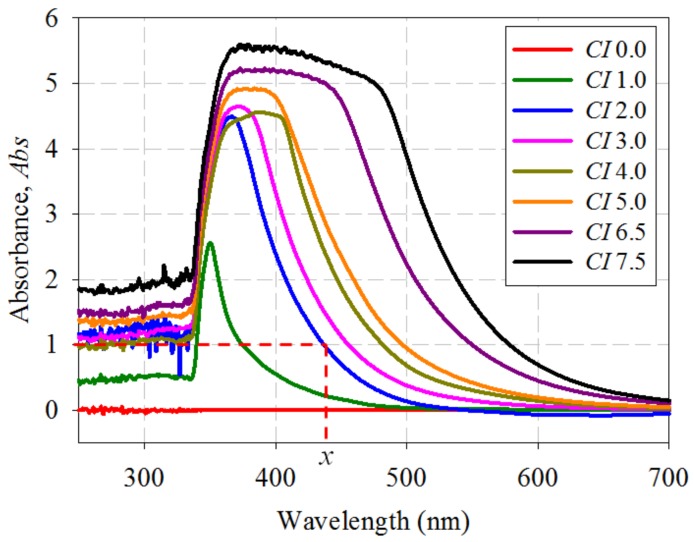
Optical absorbance spectrum of transformer oils with increasing *CI* based on ASTM D 1500.

The oil samples were re-measured with the application of the ND filter. Figure 3 shows the absorbance spectral response of several oil samples with increasing *CI*.

Figure 3 shows clearly that there is a direct relationship between the absorbance spectral response of the oil samples and their *CI*. The optical bandwidth of the spectrum at a certain *Abs* will be referred as the cutoff wavelength (*CW*) throughout the paper. For instance, the dotted line across the graph and marker *x* represents the cutoff wavelength (*CW*) at *Abs* = 1.0, for oil sample with *CI* of 2.0, which is 437 nm. It can be observed that the lowest *CI* of 1.0 shows the lowest peak absorbance and the shortest *CW* at *Abs* = 1.0, while the highest *CI* of 7.5 shows the highest peak absorbance and the longest *CW* at *Abs* = 1.0. It is worth noting that the sample with *CI* of 0.0 represents the new uninhibited transformer oil and it is used as a reference only.

### 3.2. Mathematical Modeling

**Figure 4:**
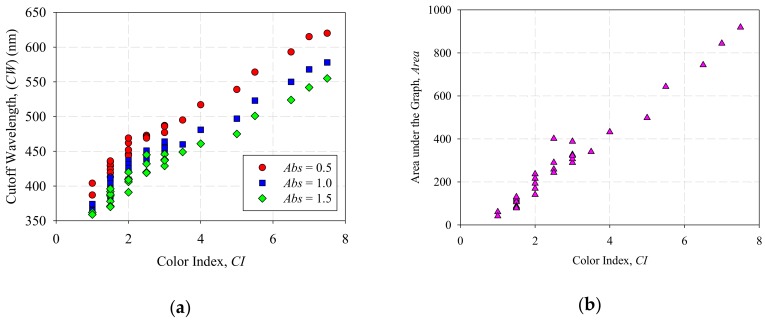
(**a**) *CW* verses *CI* of transformer oil samples at *Abs* = 0.5; 1.0 and 1.5, (**b**) *Area* verses *CI* of transformer oil samples.

Further analysis on Figure 3 shows that different variables can be used to correlate with *CI*. If a different *Abs* is chosen, the *CW* value will change accordingly. Figure 4a shows the plot of *CI* against *CW* for *Abs* = 0.5, 1.0 and 1.5 respectively. Referring to [21], Hadjadj et al. calculates the integration of the area under the graph (*Area*) from 360 nm to 600 nm to determine the DDP value in accordance to the ASTM D 6802 [37], and then relating it with the 18 Gardner color index in accordance with ASTM D 1544 [21]. Similarly, the same method can also be applied to correlate *Area* with 16 ASTM *CI* based on ASTM D 1500. Figure 4b shows the plot of *CI* against *Area.*

Based on Figure 4a, a linear relationship can be observed between the *CI* and *CW* at *Abs* = 0.5, 1.0 and 1.5. Likewise, a linear relationship can also be observed between *CI* and *Area* in Figure 4b. In order to measure the strength of the correlation between the variables, Pearson product-moment correlation coefficient (*r*) of the data was calculated. The *r* value determines the strength and direction of the linear relationship between two variables [38]. Generally, *r* > 0 indicates a positive relationship while *r* < 0 indicates a negative relationship. Table 1 shows a guideline in determining the strength of relationship for absolute value of *r* [39].

The calculated *r* values between *CI* and *CW* in Figure 4a are 0.9867, 0.9799 and 0.9772 for *Abs* = 0.5, 1.0 and 1.5 respectively. Based on Figure 4b, the calculated *r* between *CI* and *Area* is 0.9835. All four correlation coefficients are very close to 1 and it shows a very strong positive linear correlation. Thus, linear regression models can be formed based on the data in Figure 4a,b. However, it is worth noting that different linear models could be formed if different *Abs* is chosen for data in Figure 4a. Therefore a model that describes the relationship between *CI*, *CW* and *Abs* was formulated.

A 3-dimentional plot was generated to study the relationship between *CI*, *CW* and *Abs*. Three regression methods, which were Linear, Paraboloid and Gaussian regressions, were applied to the collected data to determine the method that provides the best fit. To evaluate the performance of the regression methods, the coefficient of determination (*R^2^*), adjusted *R^2^*, and standard error of estimate (*S*) were calculated. *R^2^* provides a descriptive measure of how well the regression line makes the prediction [38] while the adjusted *R^2^* is a modified version of *R^2^* that has been adjusted for the number of predictors in the model [40]. The range of both *R^2^* and adjusted *R^2^* are between 0 and 1, where 0 indicates that the measured data is far from the regression line while 1 indicates that all measured data is on the regression line. *S* measures the average distance that the measured data fall from the regression line. A smaller *S* value generally indicates that the data are closer to the regression line. Table 2 shows the analysis results of the three regression methods used.

**Figure 5:**
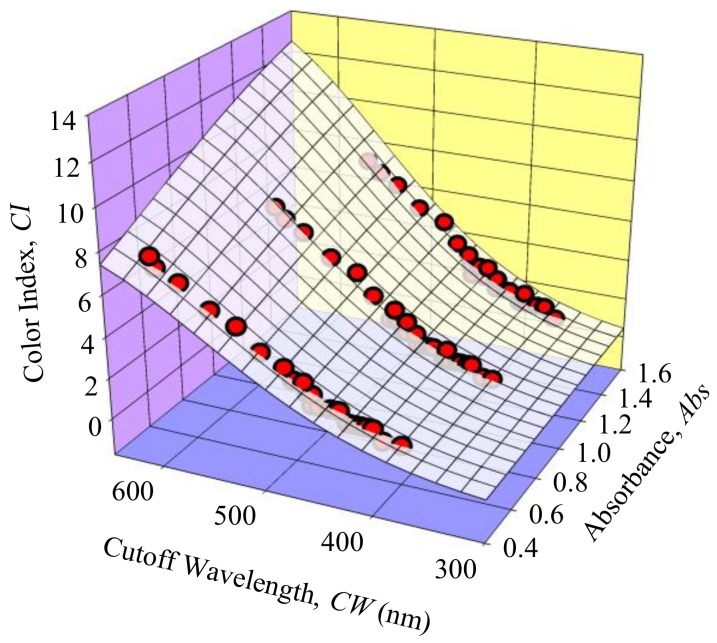
Graph of *CW* vs. *CI* of transformer oil samples vs. *Abs* (Red Circles with black borders) with Gaussian regression (White plane).

Based on Table 2, Gaussian regression shows the highest *R^2^* and adjusted *R^2^*, and the lowest *S* value compared to linear and paraboloid regressions. Therefore, Gaussian regression was chosen for the mathematical model. Figure 5 shows a 3-dimentional plot of *CW* vs. *CI* vs. *Abs* of the transformer oil with the Gaussian regression plane.

According to the results of the Gaussian regression analysis, the mathematical model that describes the relationship between the *CW*, *CI* and *Abs* of transformer oil is as Equation (2).

(2) $$CI=14.4682e^{-0.5\left[{\left(\frac{Abs\;-\;2.1818}{1.642}\right)}^{2}\;+{\left(\frac{CW\;-\;714.2849}{157.9937}\right)}^{2}\right]}.$$

**Figure 6:**
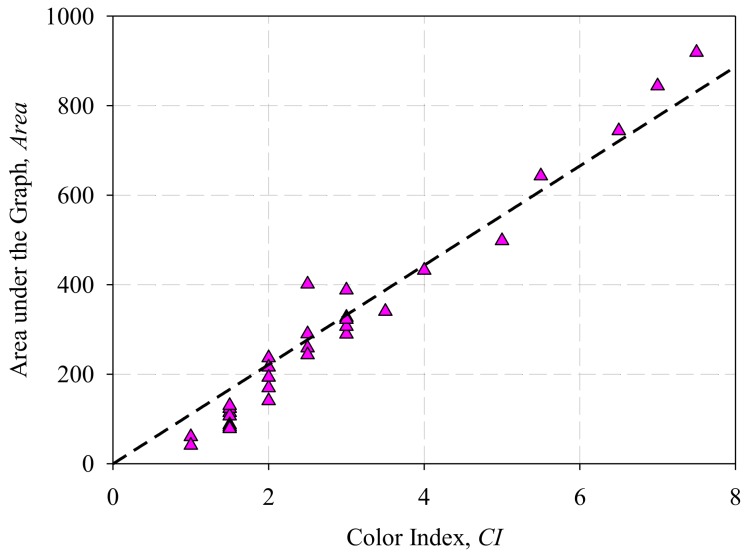
*Area* vs. *CI* of transformer oil samples with linear regression line (dotted line).

Furthermore, linear regression was applied to the collected data in Figure 4b to describe the relationship between *CI* and *Area*, and it is plotted in Figure 6.

According to the results of the linear regression analysis, the regression is able to produce a *R^2^* value of 0.9412, adjusted *R^2^* value of 0.9412, and *S* value of 55.8578. The mathematical model that describes the relationship between the *Area* and *CI* of transformer oil is as Equation (3).

(3) $$CI=\left(9.017\;\times \;10^{-3}\right)\;Area,$$

### 3.3. Verification of Mathematical Modeling

To validate the mathematical models of Equations (2) and (3), a second set of transformer oil samples were sampled. The oil samples were then sent for conventional lab analysis to determine the color index in accordance to ASTM D 1500 standard. The optical absorbance of the oil samples was also measured using Cary 5000.

For the verification of Equation (2), the *CW* of each absorbance spectral response of the oil samples at *Abs* values of 0.25, 0.5, 0.75, 1.0, 1.25, 1.5, 1.75 and 2.0 were collected, and the estimated *CI* were calculated using Equation (2). The difference between the measured *CI* and the calculated *CI* were then calculated and analyzed to determine the average difference and maximum absolute difference at different *Abs* values. Table 3 shows an example of the calculated *CI* using Equation (2), *CI* measured using ASTM D 1500 and their differences for each sample at *Abs* = 0.75.

**Figure 7:**
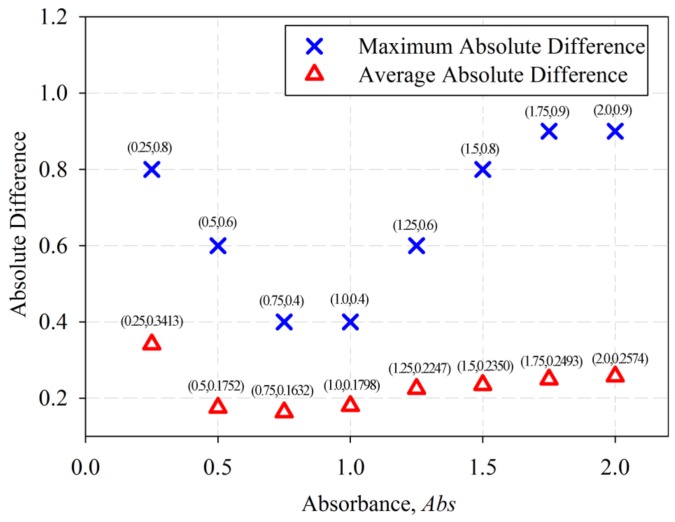
Plot of average absolute difference and maximum absolute difference at different *Abs* value.

Based on Table 3, the results show that the calculated *CI* is close to the measured *CI* value for each sample. The standard deviation and standard error for *CI* = 0.5, 1.0, 1.5, 2.0, 2.5 and 3.0 were calculated. The maximum absolute difference between the calculated and measured *CI* is 0.4 and the average absolute difference is 0.1632. The maximum absolute difference between the calculated and measured *CI*, and the average absolute difference at different *Abs* values were calculated and plotted in Figure 7.

Based on Figure 7, *Abs* of 0.75 produced the least average absolute difference (0.1632), and the *Abs* of 0.75, and 1.0 produced the lowest maximum absolute difference (0.4).

For the verification of Equation (3), the area under the graph from 360 nm to 600 nm for the second set of oil samples were recorded and the estimated *CI* were calculated using Equation (3). Likewise, the difference between the measured *CI* and the calculated *CI* were then calculated and analyzed to determine the average difference and maximum absolute difference. Table 4 shows the calculated *CI* using Equation (3), *CI* measured using ASTM D 1500 and their differences for each sample.

Based on Table 4, the results show that the calculated *CI* is close to the measured *CI* value for each sample. The standard deviation and standard error were also calculated for *CI* = 0.5, 1.0, 1.5, 2.0, 2.5 and 3.0. The maximum absolute difference between the calculated and measured *CI* is 1.8 and the average absolute difference is 0.4647.

For further verification, the Root Mean Square Error (*RMSE*) for results in Table 3 and Table 4 are calculated and compared. Generally, *RMSE* is the standard deviation of the difference between the actual value, *y* and the estimated value, *ŷ* as shown in Equation (4) [41]. *RMSE* is able to measure how much the actual data varies around the regression line.

(4) $$RMSE=\sqrt{\frac{1}{n}\overset{n}{\underset{i\;=\;1}{\sum }}{\left(y_{i}-{ŷ}_{i}\right)}^{2}},$$

where n is the total number of samples.

In this analysis, *y* represents the measured *CI* value, and *ŷ* represents the calculated CI value. *RMSE* values were calculated for both verification data in Table 3 and Table 4. Table 5 summarizes the maximum absolute differences, average absolute differences and *RMSE* values for both Equations (2) and (3).

## 4. Discussion

As described in Section 2.2, the oil samples were measured for at least 3 times to ensure that the spectrophotometer can produce consistent and repeatable results. Based on the obtained optical absorbance spectrum, the standard deviation among the calculated *CI* for each oil sample was computed. For all the samples that were measured for their repeatability, it was found that the standard deviation was in the range of 0 to 0.013.

In addition, for a particular *CI*, five different oil samples were measured and the standard deviation and standard error were calculated, as shown in Table 3 and Table 4. Due to the lack of oil samples with *CI* > 3.0 from operating power transformers, only one or two samples with *CI* > 3.0 were measured. The difficulty in getting oil samples with *CI* > 3.0 was because oil samples with *CI* > 3.0 indicate a critical level in other properties such as acidity and interfacial tension, thus the oil will be sent for oil reclamation to restore the quality of the transformer oil. Nevertheless, for *CI* ≤ 3.0, the standard deviation and standard error were calculated. Based on Table 3, the standard deviation ranges from 0.0733 to 0.2238, with the maximum percentage of standard deviation from the average value of 13%. It is important to note that not all the oil samples for this experiment were chosen such that their *CI* were determined with direct matching to the color disk. This means that the color of the oil samples could be intermediate between two standard ASTM colors. Moreover, considering that the conventional method could also cause an error of 0.5 due to its large step size, the measured *CI* also has an error of 0.5. Even with a large error, the regression is able to produce a maximum absolute difference of 0.4, and an average absolute difference of 0.1632. However, the modeled equation can be further improved by inputting more training samples with wider range of *CI*.

Results from Table 5 suggested that the determination of *CI* using Equation (2) should be done using the *CW* at *Abs* of 0.75 in order to obtain the lowest maximum absolute difference of 0.4, the least average absolute difference of 0.1632, and the smallest *RMSE* value of 0.1961. On the other hand, the results show that Equation (3) produces *CI* with a maximum absolute difference of 1.8, an average absolute difference of 0.4647 and *RMSE* value of 0.6274.

It is clear that using optical spectroscopy to determine the *CI* of the transformer oil will provide significant advantages over the conventional method. It is able to provide a smaller error due to higher resolution while the conventional method produces an error of 0.5. Moreover, the proposed technique does not require a human observer to measure the *CI*, and thus human error is eliminated.

**Figure 8:**
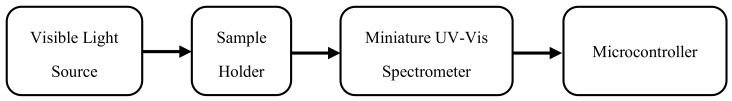
Simple block diagram of potential portable measuring prototype.

In addition, there is also a possibility that this technique can be applied and developed into a small portable handheld measuring device for on-site measurement or online monitoring. Since the interested range of wavelength is within the visible light region, a miniature UV-Vis spectrometer with a visible light source can be easily obtained at a reasonably low price. With a sample holder, a simple measuring prototype can be built. A microcontroller can also be incorporated for data processing and users interface. Figure 8 shows a simple block diagram of the measuring prototype. This prototype can be readily used by a maintenance operator for on-site *CI* measurement by extracting 3 mL of oil sample, inserting it in a cuvette, and placing the cuvette in the prototype. Besides transformer oil, the prototype can also be used for quick color measurement of other oils such as engine oil, biodiesel oil, and olive oil.

## 5. Conclusions

This work was carried out to explore the optical properties of transformer oil using optical detection. It focuses on the determination of *CI* of the transformer oil using optical spectroscopy. The existing method of determining *CI* of the transformer oil was first researched and it was found that the technique has accuracy issues, and relies on manual observations. The existing technique has the disadvantages of causing an error of 0.5 during measurement due to its large measurement step size, and the requirement of a human observer. Therefore, this paper proposes a new optical technique to quantify the *CI* of transformer oil.

Results have shown that there is a strong, positive linear correlation between the *CW* of the spectral response at a certain *Abs* and the *CI* of the transformer oil, and also between area under the curve from 360 nm to 600 nm and the *CI* of the transformer oil. Two mathematical models were formed and verified using a second set of oil samples. It was found that the equation that describes the relationship between *CW* of the spectral response at a certain *Abs* and the *CI* of the transformer oil shows smaller maximum absolute difference of 0.4, average absolute difference of 0.1632, and *RMSE* value of 0.1961 at *Abs* = 0.75.

This proposed new technique of determining the color index of transformer oil has several advantages over the current method as it has the possibility to be performed on-site, and has a higher accuracy by minimizing the risk of human error and having a higher resolution. It also has the potential to be applied to other products such as engine oil, biodiesel fuel and olive oil.

## Figures and Tables

**Table 1:** Guideline for interpretation of strength of relationship for absolute values of correlation.

Absolute Value of *r*, |*r*|	Strength of Relationship
0–0.19	Very Weak
0.20–0.39	Weak
0.40–0.59	Moderate
0.60–0.79	Strong
0.80–1.00	Very Strong

**Table 2:** Regression analysis results for linear, paraboloid and Gaussian.

Regression Method	*R^2^*	Adjusted *R^2^*	*S*
Linear	0.9563	0.9553	0.3670
Paraboloid	0.9651	0.9635	0.3317
Gaussian	0.9802	0.9793	0.2499

**Table 3:** Verification data for 42 oil samples (S1–S42) at *Abs* = 0.75 using Equation (2).

Sample	Measured *CI* ^1^	Measured Cutoff Wavelength (nm)	Calculated *CI* ^2^	Difference in *CI* ^3^	Standard Deviation	Standard Error
S1	0.5	352	0.7	0.2	0.0733	0.033
S2	0.5	357	0.8	0.3		
S3	0.5	346	0.6	0.1		
S4	0.5	361	0.8	0.3		
S5	0.5	345	0.6	0.1		
S6	1.0	371	0.9	−0.1	0.1174	0.053
S7	1.0	370	0.9	−0.1		
S8	1.0	379	1.0	0.0		
S9	1.0	385	1.1	0.1		
S10	1.0	389	1.2	0.2		
S11	1.5	427	1.9	0.4	0.2238	0.100
S12	1.5	409	1.5	0.0		
S13	1.5	402	1.4	−0.1		
S14	1.5	425	1.9	0.4		
S15	1.5	425	1.9	0.4		
S16	2.0	447	2.4	0.4	0.1839	0.082
S17	2.0	447	2.4	0.4		
S18	2.0	440	2.2	0.2		
S19	2.0	440	2.2	0.2		
S20	2.0	428	1.9	−0.1		
S21	2.5	451	2.5	0.0	0.0777	0.035
S22	2.5	458	2.7	0.2		
S23	2.5	456	2.6	0.1		
S24	2.5	453	2.5	0.0		
S25	2.5	452	2.5	0.0		
S26	3.0	467	2.9	−0.1	0.1723	0.077
S27	3.0	477	3.2	0.2		
S28	3.0	477	3.2	0.2		
S29	3.0	471	3.0	0.0		
S30	3.0	464	2.8	−0.2		
S31	3.5	487	3.5	0.0	-	-
S32	3.5	495	3.8	0.3	-	-
S33	4.0	504	4.1	0.1	-	-
S34	4.0	495	3.8	−0.2	-	-
S35	4.5	511	4.3	−0.2	-	-
S36	4.5	513	4.4	−0.1	-	-
S37	5.0	523	4.8	−0.2	-	-
S38	5.5	538	5.3	−0.2	-	-
S39	5.5	536	5.2	−0.3	-	-
S40	6.5	568	6.4	−0.1	-	-
S41	7.0	582	7.0	0.0	-	-
S42	7.5	592	7.3	−0.2	-	-

^1^ Color index based on measurement in accordance of ASTM D 1500, ^2^ Color index calculated based on Equation (2), ^3^ Difference in *CI* = Calculated *CI* – Measured *CI*.

**Table 4:** Verification data for 42 oil samples (S1–S42) using Equation (3).

Sample	Measured *CI* ^1^	*Area* ^2^	Calculated *CI* ^3^	Difference in *CI* ^4^	Standard Deviation	Standard Error
S1	0.5	11.7707	0.1	−0.4	0.0480	0.021
S2	0.5	11.3844	0.1	−0.4		
S3	0.5	4.0164	0.0	−0.5		
S4	0.5	16.6769	0.2	−0.3		
S5	0.5	4.6769	0.0	−0.5		
S6	1.0	48.885	0.4	−0.6	0.0872	0.039
S7	1.0	37.4572	0.3	−0.7		
S8	1.0	46.276	0.4	−0.6		
S9	1.0	56.0387	0.5	−0.5		
S10	1.0	62.8738	0.6	−0.4		
S11	1.5	148.5237	1.3	−0.2	0.2465	0.110
S12	1.5	103.449	0.9	−0.6		
S13	1.5	83.9942	0.8	−0.7		
S14	1.5	138.809	1.3	−0.2		
S15	1.5	136.7029	1.2	−0.3		
S16	2.0	236.6137	2.1	0.1	0.3090	0.138
S17	2.0	236.3961	2.1	0.1		
S18	2.0	198.7913	1.8	−0.2		
S19	2.0	209.3838	1.9	−0.1		
S20	2.0	153.3042	1.4	−0.6		
S21	2.5	246.3	2.2	−0.3	0.1903	0.085
S22	2.5	296.6479	2.7	0.2		
S23	2.5	274.906	2.5	0.0		
S24	2.5	264.5622	2.4	−0.1		
S25	2.5	246.69	2.2	−0.3		
S26	3.0	503.8327	4.5	1.5	0.6625	0.296
S27	3.0	355.9228	3.2	0.2		
S28	3.0	374.7372	3.4	0.4		
S29	3.0	343.9266	3.1	0.1		
S30	3.0	313.9438	2.8	−0.2		
S31	3.5	435.9306	3.9	0.4	-	-
S32	3.5	580.9014	5.2	1.7	-	-
S33	4.0	538.9002	4.9	0.9	-	-
S34	4.0	430.8067	3.9	−0.1	-	-
S35	4.5	547.1639	4.9	0.4	-	-
S36	4.5	345.1915	3.1	−1.4	-	-
S37	5.0	572.1769	5.2	0.2	-	-
S38	5.5	805.9143	7.3	1.8	-	-
S39	5.5	624.9499	5.6	0.1	-	-
S40	6.5	743.9695	6.7	0.2	-	-
S41	7.0	821.1863	7.4	0.4	-	-
S42	7.5	901.6618	8.1	0.6	-	-

^1^ Color index based on measurement in accordance of ASTM D 1500, ^2^ Integration of area under the graph from 360 nm to 600 nm, ^3^ Color index calculated based on Equation (3), ^4^ Difference in *CI* = Calculated *CI* – Measured *CI*.

**Table 5:** Maximum absolute difference, average absolute difference, and *RMSE* value for verification data.

	*Abs*	Maximum Absolute Difference	Average Absolute Difference	*RMSE*
**For Equation (2)**	0.25	0.8	0.3413	0.3972
	0.50	0.6	0.1752	0.2235
	0.75	0.4	0.1632	0.1961
	1.00	0.4	0.1798	0.2144
	1.25	0.6	0.2247	0.2747
	1.50	0.8	0.2350	0.2981
	1.75	0.9	0.2493	0.3163
	2.00	0.9	0.2574	0.3249
**For Equation (3)**	-	1.8	0.4647	0.6274
